# Case Series: Efficacy and Safety of Hemoadsorption With HA-330 Adsorber in Septic Pediatric Patients With Cancer

**DOI:** 10.3389/fped.2021.672260

**Published:** 2021-06-11

**Authors:** Vitaliy Sazonov, Ramazan Abylkassov, Zaure Tobylbayeva, Askhat Saparov, Olga Mironova, Dimitri Poddighe

**Affiliations:** ^1^Department of Biomedical Sciences, Nazarbayev University School of Medicine, Nur-Sultan, Kazakhstan; ^2^Pediatric Anesthesiology and Intensive Care Unit, National Research Center for Maternal and Child Health, “University Medical Center”, Nur-Sultan, Kazakhstan; ^3^Department of Medicine, Nazarbayev University School of Medicine, Nur-Sultan, Kazakhstan; ^4^Department of Pediatrics, National Research Center for Maternal and Child Health, “University Medical Center”, Nur-Sultan, Kazakhstan

**Keywords:** sepsis, pediatric cancer, blood purification, hemoadsorption, HA-330 adsorber

## Abstract

**Background:** Sepsis is a frequent cause of death in hospitalized patients and, in detail, in neonatal, pediatric, and adult intensive care units (ICUs). Severe sepsis has a very poor prognosis. Indeed, the mortality rate varies between 30 and 70% during the first 7–14 days. Despite a timely and appropriate therapy, the prognosis of severe sepsis is too often negative. Therefore, new therapeutic resources are under investigation in order to further improve prognosis.

**Case series:** Here, we reported three septic children in whom we used extracorporeal blood purification therapy with hemoadsorption device HA330 (Jafron Biomedical Co., Ltd., China), aiming to scavenge and eliminate bacterial toxins and inflammatory mediators from the blood.

**Discussion and Conclusion:** This small case series first showed that hemoperfusion with HA330 cartridge may be an effective and relatively safe adjunctive treatment to counterbalance the cytokine storm in septic children with hematological disorders. Further studies are needed to confirm and further support its safety and efficacy in a large number of pediatric patients.

## Introduction

Sepsis is a frequent cause of death in hospitalized patients and, in detail, in neonatal, pediatric, and adult intensive care units (ICUs). According to WHO, almost 49 million people were hospitalized with sepsis worldwide in 2017, and 11 million patients died; importantly, almost half of these hospitalized patients were children under 5 years of age (1). According to a recent systematic review with meta-analysis, sepsis incidence and prevalence were estimated to be 48 per 100,000 persons-year and 22 per 100,000 people, respectively (2).

Sepsis can lead to life-threatening multiple organ dysfunction due to a dysregulated immunologic and metabolic host response to an infection (3). In pathophysiological terms, sepsis can be defined as a dysregulated systemic inflammatory response syndrome (SIRS) associated with an infection (usually bacterial). Clinically, SIRS is diagnosed by the presence of at least two of the following criteria (including one of the first two, mandatorily): abnormal temperature (>38.5 or <36°C), abnormal leukocyte count (leukocytosis or leukopenia for age-appropriate reference values), tachycardia [>2 standard deviations (SDs) above the normal age-related values], tachypnea (>2 SD above the normal age-related values). In severe sepsis, children develop cardiovascular (systolic blood pressure <-2 SD for age and need for vasoactive drug, which correspond to the definition of septic shock) or respiratory (PaO_2_/FiO_2_ <300 with need for mechanical ventilatory support) insufficiency, along with multiple organ dysfunction. Severe sepsis has a very poor prognosis. Indeed, the mortality rate varies between 30 and 70% during the first 7–14 days (4, 5).

The pathophysiological mechanisms of sepsis are very complex and have not been completely elucidated yet. Once the causing pathogen enters the bloodstream, if it cannot be appropriately and timely cleared for several and variable reasons, bacterial toxins may lead to the uncontrolled production of a cascade of pro-inflammatory cytokines [including interleukin (IL)-1β, IL-6, IL-12, tumor necrosis factor (TNF)-α)] (6, 7). This “cytokine storm” progressively damages several tissues and organs and, importantly, creates an “endothelial dysfunction,” which alters the balance between the coagulation process and fibrinolysis, and finally results in the aggravation of tissue hypoperfusion, leading to an irreversible and multiple organ dysfunction (6). Indeed, septic patients are often affected by underlying diseases that cause host immunodepression. For instance, sepsis can complicate the clinical picture of patients affected with hematological malignancies, as it is in our case series.

In general, the mainstay of sepsis treatment in children consists of antibiotic and supportive therapies (including intravenous fluid replacement, mechanical ventilation, cardiotonic drugs, etc.). Unfortunately, the prognosis of severe sepsis is too often negative, despite a timely and appropriate therapy, as previously mentioned. Therefore, new therapeutic resources are under investigation in order to further improve prognosis. Here, we reported three septic children in whom we used extracorporeal blood purification therapy with hemoadsorption device HA330 (Jafron Biomedical Co., Ltd., China), aiming to scavenge and eliminate bacterial toxins and inflammatory mediators from the blood.

## Clinical Cases

### Patient 1

A 6-month-old girl was previously diagnosed with pure red cell aplasia. She developed fever, anuria, and progressive skin necrosis on the abdominal wall and buttock areas. Based on clinical (persistent hyperthermia, hypotension, oliguria), laboratory (leukocytosis and elevated inflammatory parameters), and microbiological (blood cultures positive for *Staphylococcus epidermidis*) findings, this patient was diagnosed with sepsis and transferred to the pediatric intensive care unit (PICU). Despite the antibiotic therapy (meropenem, amikacin, metronidazole) and the supportive therapy, her clinical conditions gradually worsened and, on day 10 after the PICU admission, the patient also needed respiratory support [non-invasive ventilation (NIV)] and showed a drastic increase of plasmatic urea/creatinine and all the inflammatory markers. Therefore, this clinical condition of acute kidney injury led to the initiation of pediatric continuous venovenous hemodiafiltration (CVVHDF) with the “Prismaflex” device (Baxter, US) (with the following prescription parameters: flow rate, 50 ml/min; both pre-dilution and post-dilution, −150 ml/h; ultrafiltration, 20 ml/h; prolonged heparinization, 5–30 IU/kg/h; effluent, 600 ml/h; dialysate fluid, 300 ml/h) by using disposable hemoperfusion cartridge HA 330 (Jafron Biomedical Co., Ltd., China) for 4 h. An appropriate (for child's size) hemodialysis catheter was inserted into the right subclavian vein. Normal saline was used for the priming of the circuit and system testing; before connecting the device to the patient, normal saline was replaced with red blood cell suspension.

After this first session of CVVHDF, all renal and inflammatory parameters rapidly improved, as summarized in Table 1. Importantly, the patient was also able to wean from the ventilatory support, and the FiO_2_ decreased from 60 to 30%; moreover, catecholamines were gradually discontinued. However, 5 days after the above treatment (day 15), the patient's clinical condition worsened again. Therefore, a second session of CVVHDF with HA-330 was performed, which resulted in similar improvements as reported in the previous episode (as shown in Table 1). No further CVVHF sessions were required, and the patient was discharged from the PICU 2 weeks later (day 29).

**Table 1:** Main clinical and laboratory parameters (patient 1).

**Parameter**	**At the PICU admission**	**Start (before the first procedure)**	**After the first procedure**	**Reduction rate, % (if applicable)**	**Start (before the second procedure)**	**After the second procedure**	**Reduction rate, % (if applicable)**	**At the discharge from PICU**
Leukocytes, ^*^10^9^/L	12.20	30.00	5.36	−82.13	20.27	3.37	−83.37	5.39
Neutrophils, %	88.10	85.20	78.12	−8.31	69.91	71.11	1.72	61.45
Hemoglobin, g/L	70.00	113.00	111.00	−1.77	131.00	121.00	−7.63	123.00
Thrombocytes	118.00	119.00	131.00	10.08	181.00	178.00	−1.66	204.00
CRP, mg/mL	396.14	517.09	138.62	−73.19	350.75	5.27	−98.50	24.18
Procalcitonin, ng/ml	12.13	20.28	4.27	−78.94	71.28	4.77	−93.31	2.21
S100 protein, μmol/L	N/A	3.54	0.13	−96.33	25.18	0.12	−99.52	N/A
IL-6, pg/ml	N/A	839.00	67.74	−91.93	1,845.00	20.35	−98.90	N/A
Troponin, pg/ml	N/A	108.30	84.71	−21.78	197.70	36.50	−81.54	N/A
Creatine kinase (CK), IU/L	N/A	68.00	26.00	−61.76	27.00	10.00	−62.96	N/A
ALT, IU/L	34.54	40.04	34.01	−15.06	46.2	12.40	−73.16	21.31
AST, IU/L	31.21	24.79	24.57	−0.89	40.10	10.80	−73.07	18.75
BUN, mmol/L	23.91	36.14	11.67	−67.71	5.30	2.60	−50.94	3.1
Creatinine, μmol/L	76.31	92.59	32.27	−65.15	13.00	14.00	7.69	10.00
pH	7.291	7.053	7.349		7.352	7.380		7.333
cBase(Ecf), c, mmol/L	−3.3	−6.7	−2.6		−3.9	4.0		−1.3
${\text{HCO}}_{3}^{-}$, mmol/L	19.21	16.9	23.6		27.4	28.0		26.1
pCO_2_, mmHg	43.4	106.0	45.3		42.0	38.5		41.1
pO_2_, mmHg	73.3	54.7	68.9		69.7	75.5		77.1
Dopamine, μg/kg/min	–	6	–		5	–		–
NIBP, mmHg	68/41	57/36	97/36		68/42	94/52		98/54
Ventilation	O_2_ flow, 2 L/min	NIV, FiO_2_ – 60%, Psupp. – 3, PEEP – 5.0	Spont mode, FiO_2_ 30%		NIV, FiO_2_ – 50%, Psupp. – 3, PEEP – 5.0	Spont mode, FiO_2_ 30%		Spontaneous breathing
SpO_2_/FiO_2_	327	162	327		192	320		466.6
Diuresis, ml/kg/h	0.9	0.6	3.0		1.8	2.9		2.2

*CRP, C reactive protein; IL-6, Interleukin 6; ALT, Alanine Aminotransferase; AST, Aspartate Aminotransferase; BUN, Blood Urea Nitrogen; NIBP, Non-Invasive Blood Pressure; NIV, Non-Invasive Ventilation; FiO_2_, fraction of inspired oxygen; Psupp, pressure support; PEEP, Positive End-Expiratory Pressure; Spont mode, Spontaneous Mode of Mechanical Ventilation; HCO_2_, Bicarbonate; pCO_2_, Partial Pressure of Carbon Dioxide; pO_2_, Partial Pressure of Oxygen; SpO_2_/FiO_2_, Oxygen Saturation to Fraction of Inspired Oxygen Ratio; cBase(Ecf), Base Excess in Extracellular Fluid; HPAP, Hooded/Helmet Positive Airway Pressure; APVSIMV, Synchronized Intermittent Mandatory Ventilation with Adaptive Pressure Ventilation; SIMV, Synchronized Intermittent Mandatory Ventilation*.

### Patient 2

A 14-year-old boy diagnosed with drug-resistant and relapsed acute lymphoblastic leukemia (ALL) was admitted for chemotherapy according to FLAG-IDA protocol (fludarabine, cytarabine, idarubicin) (8). On the eighth day after completing the chemotherapy, the patient developed sepsis sustained by (extended-spectrum β lactamase-producing) *Escherichia coli*. Because of the development of multiorgan failure (including anuria and respiratory failure), he was transferred to the PICU.

Despite the antibacterial treatment (trimethoprim/sulfamethoxazole, piperacillin-tazobactam, and amikacin), the patient showed no improvement. Due to persistent fever (associated with high and increasing values of the inflammatory markers) and constantly high levels of plasmatic urea along with anuria (despite the high dose of furosemide, 3 mg/kg/day) on day 7 from PICU admission, he underwent CVVHDF (with the following prescription parameters: flow rate, 120 ml/min; both pre-dilution and post-dilution, −800 ml/h; ultrafiltration, 60 ml/h; prolonged heparinization, 5–30 IU/kg/h; effluent, 3,200 ml/h; dialysate fluid, 1,600 ml/h) with HA 330 cartridge for 4 h. At the end of this procedure (as summarized in Table 2), this patient's inflammatory and renal parameters significantly improved, as well as his respiratory function. Interestingly, all the inflammatory markers decreased, except for S100 protein. The patient was weaned from mechanical ventilation on day 9 and achieved hemodynamic stability without any catecholamines on day 10. He was discharged from the PICU on day 12.

**Table 2:** Main clinical and laboratory parameters (patient 2).

**Parameter**	**At the PICU admission**	**Start (before the procedure)**	**After the procedure**	**Reduction rate, % (if applicable)**	**At the discharge from PICU**
Leukocytes, ^*^10^9^/L	0.03	0.01	0.03	200.00	0.03
Neutrophils	N/A	N/A	N/A		N/A
Hemoglobin, g/L	73.00	101.00	102.00	0.99	98.00
Thrombocytes, ^*^10^9^/L	1.00	1.00	3.00	200.00	1.00
CRP, mg/L	138.62	378.02	131.29	−65.27	12.28
Procalcitonin, ng/ml	23.12	103.04	9.14	−91.13	1.91
S100 protein, μg/L	N/A	0.27	0.26	−3.70	N/A
IL-6, pg/ml	N/A	352.20	184.30	−47.67	N/A
Creatine kinase (CK), IU/L	N/A	6.00	5.00	−16.67	N/A
ALT, IU/L	81.35	277.00	160.90	−41.91	67.90
AST, IU/L	24.57	216.00	58.40	−72.96	35.39
BUN, mmol/L	5.33	17.30	8.07	−53.35	9.26
Creatinine, μmol/L	34.77	138	67	−51.45	21
pH	7.420	7.041	7.501		7.415
cBase (Ecf), c, mmol/L	0	−5	2		1
${\text{HCO}}_{3}^{-}$, mmol/L	24.9	29	25		26
pCO_2_, mmHg	38.2	53	35		41
pO_2_, mmHg	31	72	80		78
Dopamine, μg/kg/min	–	5	–		–
NIBP, mmHg	94/51	85/50	95/60		101/50
Ventilation	O_2_ flow, 2 L/min	HPAP, FiO_2_ – 60%	–		–
SpO_2_/FiO_2_	320	158	466		461
Diuresis, ml/kg/h	–	–	3		2.7

### Patient 3

A 2.5-year-old girl diagnosed with ALL was admitted for chemotherapy with high-dose methotrexate according to ALL BFM IC 2002 Block HR1 protocol (9). After the 24-h high-dose methotrexate infusion (HD-MTX), she developed severe toxic mucositis, epidermolysis, and hepatitis.

Therefore, this patient was transferred to the PICU. On day 4, she developed sepsis characterized by acute kidney injury and bilateral pneumonia, even though no clear microbiological agents were identified. Indeed, the blood culture samples were obtained during antibiotic therapy. The diagnosis of sepsis was made on the basis of the clinical picture and a high level of procalcitonin (848 ng/L). Despite the antibacterial treatment with cefixime and amikacin, no improvement was noticed, and the patient required respiratory support with mechanical ventilation [synchronized intermittent mandatory ventilation with adaptive pressure ventilation (APVSIMV) mode]. Thus, the patient underwent CVVHDF (with the following prescription parameters: flow rate, 80 ml/min; both pre-dilution and post-dilution, −500 ml/h; ultrafiltration, 100 ml/h; prolonged heparinization, 5–30 IU/kg/h; effluent, 2,000 ml/h; dialysate fluid, 1,000 ml/h) with HA-330 cartridge for 4 h on day 7. After the procedure, the patient's general condition and laboratory parameters (Table 3) improved significantly. Importantly, the respiratory support was switched to synchronized intermittent mandatory ventilation (SIMV), and her dopamine requirement decreased twice compared to the previous days. In addition, there was good clinical dynamics and healing of necrotic skin sites.

**Table 3:** Main clinical and laboratory parameters (patient 3).

**Parameter**	**At the PICU admission**	**Start (before the procedure)**	**After the procedure**	**Reduction rate, % (if applicable)**
Leukocytes, ^*^10^9^/L	10.81	16.01	8.03	−49.84
Neutrophils, %	59.98	96.08	88.87	−7.50
Hemoglobin, g/L	106.00	95.00	101.60	6.95
Thrombocytes, ^*^10^9^/L	147.00	203.00	213.00	4.93
CRP, mg/L	110.55	259.40	130.03	−49.87
Procalcitonin, ng/L	28.41	848.00	49.60	−94.15
S100 protein, μg/L	N/A	1.45	0.26	−82.07
IL-6, pg/ml	N/A	191.30	66.03	−65.48
Creatine kinase (CK), IU/L	N/A	8.00	4.00	−50.00
ALT, IU/L	124.90	28.64	12.80	−55.31
AST, IU/L	209.56	97.24	59.40	−38.91
BUN, mmol/L	6.64	26.49	8.05	−69.61
Creatinine, μmol/L	87.07	178.11	65.00	−63.51
cBase (Ecf), c, mmol/L	−6.2	−5	2	
${\text{HCO}}_{3}^{-}$, mmol/L	14	16	23	
pCO_2_, mmHg	32.9	62	48	
pO_2_, mmHg	34.2	64	78	
Dopamine, μg/kg/min	–	10	5	
Norepinephrine, μg/kg/min	–	0.2	–	
NIBP, mmHg	101/70	65/35	92/60	
Ventilation	O_2_ flow, 4 L/min	APVSIMV	SIMV	
Oxygen saturation index (OSI)	(SpO_2_/FiO_2_ – 274)	7.8	4.2	
Diuresis, ml/kg/h	1.4	0.8	2.9	

Unfortunately, despite such a positive response to CVVHDF procedure, the patient had a fatal outcome on day 28 (namely, 21 days after the CVVHDF session) because of the underlying hematologic malignancy, which became drug-resistant.

## Discussion

Sepsis is a frequent cause of PICU admission and mortality for children affected with leukemia and, in general, severe hematologic diseases (10, 11). In detail, Aljabari et al. (12) still reported high rates of morbidity and mortality among this group of patients despite the improvements in supportive care and microbiological treatment: 8% of children develop severe sepsis requiring treatment in the PICU, and 34% of them die or develop multiple organ dysfunction syndrome. Therefore, additional strategies against sepsis should be sought. One of these therapeutic resources is CVVHDF through disposable hemoperfusion cartridge HA 330.

There are different techniques for extracorporeal blood purification used during a cytokine storm, such as high-volume hemofiltration (HVHF), plasma exchange (PE), high-cut-off (HCO) membrane, hemoadsorption (with filters and adsorption columns, including selective polymyxin B, nonselective CytoSorb, HA, etc.). To date, several papers on the use of HVHF in children with sepsis have been published; some authors concluded that such a procedure can improve the outcome of sepsis (13). Actually, Miao et al. (14) reported no significant difference on 28-day mortality, improvement of hemodynamic profile, and clearing of inflammatory factors in critically ill pediatric patients with severe sepsis when using HVHF compared to the standard-volume continuous venovenous hemofiltration (14). Moreover, other authors were against HVHF over standard hemofiltration in children with septic shock or sepsis-associated organ dysfunction (15). PE can potentially improve organ function in septic patients; however, a multicenter retrospective study including patients younger than 18 years did not show any decrease in mortality in both patients with continuous renal replacement therapy and PE usage (16). Additionally, PE can lead to a deficiency of coagulation factors and hypoalbuminemia, in addition to further risk of infections. Regarding HCO, the review by Ankawi et al. (17) concluded that there is no evidence to support its use in sepsis, although a study in adults suggested a decrease in inflammatory cytokines and improvement of hemodynamics (18).

HA330 is characterized by a hemoperfusion cartridge with an electrically porous resin used specifically to remove cytokines, complements, and other endotoxins with molecular weight of 10–60 kDa. It is used primarily during acute and severe clinical conditions associated with a cytokine storm, as it can occur during sepsis (19). This cartridge was also effective in the treatment of hepatitis (decreased levels of IL-8, ammonia, bilirubin) and pancreatitis (decreased level of lipids and amylase) (19). Conversely, the Prismaflex hemofilter used for CVVHDF eliminates low- and medium-molecular weight compounds and only partially eliminates beta 2-microglobulinin (12 kDa), TNF-α (17 kDa), IL-6 (26 kDa), and IL-10 (30 kDa) (20). For this reason, a CVVHDF session alone is not usually effective to eliminate these inflammatory mediators. Therefore, the appropriate selection of cartridges can play a significant role in the treatment of specific clinical conditions. For example, the elimination of endotoxins is best carried out by selective polymyxin B, rather than a non-selective cartridge, because their molecular weight cutoff point is higher than the non-selective (~100 vs. ~60 kDa, respectively) (21). HA330 and CytoSorb are very similar cartridges functionally; however, CytoSorb is the only approved extracorporeal technique in European Union, whereas HA330 is mainly used in China (21).

Our previous experience with CytoSorb showed a 32% reduction rate of IL-6 (22). Here, HA330 appeared to be more effective in eliminating IL-6 from the bloodstream compared to CytoSorb. The issue of the effectiveness of Cytosorb in reducing the plasmatic levels of inflammatory cytokines remains controversial based on the available publications. According to Schadler et al. (23), CytoSorb did not reach a statistically significant decrease in IL-6 blood levels when compared to the control group with no hemoperfusion. Conversely, a recent study done by Bottari et al. (24) showed a significant reduction of Il-6 and IL-10 by using Cytosorb with continuous renal replacement therapy as blood purification strategy in pediatric septic shock.

To date, hemoadsorption using the HA-330 cartridge has been well-studied in adult patients with inflammatory conditions such as sepsis, acute lung injury, hepatitis, and pancreatitis (19). In these clinical settings, a marked reduction of inflammatory mediators, noticeable clinical improvement, and, importantly, no significant side effects were reported (19). Moreover, several reports suggested some benefits from this procedure even in patients affected with septic shock (17, 25–27). However, large clinical studies on the use of extracorporeal methods in pediatric sepsis are currently missing (17). Therefore, the indication and choice of specific extracorporeal methods, such as hemofiltration or hemoadsorbtion, in septic patients can be based on the clinical assessment of individual clinical cases.

To our knowledge, here we reported the first small case series of septic children treated with CVVHDF by using HA330 cartridge. In all our three cases, we noted a combination of acute kidney injury (combined with or without multiple organ failure), which was the reason for initiating the pediatric CVVHDF by using the Prismaflex hemofiltration system and poly membrane (AN69) filters. The HA-330 adsorber was installed after the hemofilter. Heparin titration rate was corrected on the basis of the activated partial thromboplastin time and maintained at 60–80 seconds. In all these cases, normal saline was used for the priming of the circuit and system testing; however, in two cases (Patient 1 and Patient 3), before connecting the device to the patient, normal saline was replaced with red blood cell suspension. Before the procedure, double-lumen central venous catheters were percutaneously placed to the right subclavian vein under sedation and analgesia; the correct location was confirmed by chest X-ray.

The response to the treatment was positive in all three cases based on inflammatory markers (as summarized in Table 4) and clinical improvements (including the weaning from the ventilatory support and vasoactive drugs). Despite the small number of procedures performed (*n* = 4, in one patient, the procedure was performed twice), we noted a remarkable decrease in all the inflammatory markers. In all these patients, the antibiotic therapy alone did not seem to be enough, but the CVVHDF with disposable hemoperfusion cartridge HA 330 administered in 4-h sessions apparently rescued—or contributed to rescue at least—the clinical situation: they experienced a stabilization of hemodynamics with half of the pretreatment dosage to none at all. As for the lung function, after hemoperfusion, these patients required much milder regimens of mechanical ventilation to none at all, and their blood gas values returned to age-appropriate normal ranges. As recommended by the Pediatric Acute Lung Injury Consensus Conference Group, the improvement of the respiratory failure was described by showing the variations in time of the S/F ratio or oxygen saturation index (28). Moreover, all children also showed remarkable improvement of diuresis and kidney function tests, as well as a decrease of transaminases, if altered.

**Table 4:** Plasma concentrations of CRP, procalcitonin, S100 protein, and IL-6 before and after blood purification procedure (mean ± SD, *n* = 4).

	**Before**	**After**	**% of reduction**
CRP, mg/L	376.32 ± 106.68	101.30 ± 64.13	−71.71 ± 20.32%
Procalcitonin, ng/L	260.65 ± 393.05	16.95 ± 21.78	−89.38 ± 7.07%
S100 protein, μg/L	7.54 ± 11.84	0.19 ± 0.08	−69.24 ± 44.77%
IL-6, pg/ml	806.88 ± 744.85	84.6 ± 69.99	−75.99 ± 23.74%

Sepsis mortality is greatly affected by the development of multiorgan (cardiovascular, pulmonary, renal, etc.) failure during its clinical course (2). Moreover, patients with leukemia and sepsis showed much higher mortality rates than other general (non-hematologic) PICU patients (11). Early empiric antibiotic treatment and life-supportive therapies are crucial for the successful management of septic children (29). However, adjunctive therapies, like hemoperfusion, may greatly increase the survival rate. A randomized controlled trial by Huang et al. (25) showed a significant decrease in ICU and hospital mortality among a group of septic patients who underwent hemoperfusion with HA330 compared to that of the control group receiving standard treatment (12.5 vs. 45.0% and 37.5 vs. 50.0%, respectively) (25). This approach can effectively counteract the cytokine storm that characterizes sepsis as an additional therapeutic tool to the “conventional” immune-modulatory therapies (25, 30). In our small case series, extracorporeal purification methods seem to represent a promising adjunctive therapy for severe sepsis in children. However, this is currently the only experience with HA330-related blood purification in septic children. Therefore, additional and independent reports and, possibly, clinical studies are needed to make final conclusions on the effectiveness and safety of this procedure in children with hematological diseases and malignancies. Indeed, the typical complications of extracorporeal methods (e.g., catheter bleeding and infection, heparinization side effects, decreased platelets, etc.) can be encountered, but the shorter procedure time required by HA330-related blood purification can reduce these risks.

## Conclusion

This small case series first suggested that the hemoperfusion with HA330 cartridge may be an effective and relatively safe adjunctive treatment to counterbalance the cytokine storm in septic children affected with hematological disorders. However, further clinical studies are needed to support our first and preliminary experience.

## Data Availability Statement

The original contributions presented in the study are included in the article/supplementary material, further inquiries can be directed to the corresponding author/s.

## Ethics Statement

Ethical review and approval was not required for the study on human participants in accordance with the local legislationand institutional requirements. This case series complies with the guidelines for human studies, and the research was conducted ethically in accordance with the World Medical Association Declaration of Helsinki. Written informed consent was obtained from the patients' parents. The report is fully anonymized.

## Author Contributions

VS contributed to the literature search, collecting of data, and writing and editing of the manuscript. RA contributed to the literature search, collecting of data, and drafting of the manuscript. ZT participated in patient care and collecting of data. AS and OM contributed to collecting of data. DP contributed to writing the draft and critically revised the manuscript. All authors reviewed the draft, modified it accordingly, and approved the final version.

## Conflict of Interest

The authors declare that the research was conducted in the absence of any commercial or financial relationships that could be construed as a potential conflict of interest.
